# Chromogenic *in situ *hybridisation for the assessment of HER2 status in breast cancer: an international validation ring study

**DOI:** 10.1186/bcr1776

**Published:** 2007-10-08

**Authors:** Marc van de Vijver, Michael Bilous, Wedad Hanna, Manfred Hofmann, Petra Kristel, Frédérique Penault-Llorca, Josef Rüschoff

**Affiliations:** 1 Netherlands Cancer Institute, Amsterdam, The Netherlands; 2 Westmead Hospital, Westmead, New South Wales, Australia; 3 Sunnybrook & Women's College, Health Science Center, Toronto, Canada; 4 Klinikum Kassel, Kassel, Germany; 5 Departement de Pathology, Centre Jean Perrin, Clermont-Ferrand, France

## Abstract

**Introduction:**

Before any new methodology can be introduced into the routine diagnostic setting it must be technically validated against the established standards. To this end, a ring study involving five international pathology laboratories was initiated to validate chromogenic *in situ *hybridisation (CISH) against fluorescence *in situ *hybridisation (FISH) and immunohistochemistry (IHC) as a test for assessing human epidermal growth factor receptor 2 (HER2) status in breast cancer.

**Methods:**

Each laboratory performed CISH, FISH and IHC on its own samples. Unstained sections from each case were also sent to another participating laboratory for blinded retesting by CISH ('outside CISH').

**Results:**

A total of 211 invasive breast carcinoma cases were tested. In 76 cases with high amplification (HER2/CEP17 ratio >4.0) by FISH, 73 cases (96%) scored positive (scores ≥ 6) by 'outside CISH'. For FISH-negative cases (HER2/CEP17 ratio <2.0), 94 of 100 cases (94%) had CISH scores indicating no amplification (score ≤ 5), and only three cases were positive by CISH; in the three remaining cases, no CISH result could be obtained. For cases with low-level amplification using FISH (HER2/CEP17 ratio 2.0–4.0), 20 of 35 had CISH scores indicating gene amplification. Inter-laboratory concordance was also very high: 95% for normal *HER2 *copy number (1–5 copies); and 92% for cases with *HER2 *copy numbers ≥ 6. CISH intra-laboratory concordance with IHC was 92% for IHC-negative cases (IHC 0/1+) and 91% for IHC 3+ cases. Among IHC 2+ cases, CISH was 100% concordant with samples showing high amplification by FISH, and 94% concordant with FISH-negative samples.

**Conclusion:**

These results show that CISH inter- and intra-laboratory concordance to FISH and IHC is very high, even in equivocal IHC 2+ cases. Therefore, we conclude that CISH is a methodology that is a viable alternative to FISH in the HER2 testing algorithm.

## Introduction

Human epidermal growth factor receptor 2 (HER2) is strongly overexpressed in 20–30% of human breast cancers (the *HER2 *gene is also known as *ERBB2 *or *neu*). HER2 is a 185 kDa transmembrane growth factor receptor with tyrosine kinase activity, and has been shown to play a role in the signal transduction of cell growth [1,2]. It has been shown in many studies that overexpression of the HER2 protein correlates with amplification of the *HER2 *gene [3-7]. A HER2-positive status has been associated with a poor prognosis, including aggressive disease and shorter survival [8], and there also is evidence that HER2-positive tumours differ from HER2-negative tumours in their responsiveness to chemotherapy and hormonal therapy [9-14].

Trastuzumab (Herceptin), a humanised monoclonal antibody, has been specifically developed to target HER2. Trastuzumab has been shown unequivocally to confer a survival benefit in the treatment of women with HER2-positive breast cancer [15,16]. As responsiveness to trastuzumab therapy is directly linked to a patient's HER2 status, accurate determination of HER2 status is essential for identifying patients who may benefit from this form of treatment. It has been clearly shown that HER2-positive patients, that is, patients whose tumours show strong overexpression of HER2 as indicated by an immunohistochemistry (IHC) score of 3+, and/or whose tumours show *HER2 *gene amplification as determined by a positive fluorescence *in situ *hybridisation (FISH) result, derive the greatest therapeutic benefit from treatment with trastuzumab [15,17].

Several different techniques have been used to determine HER2 status in breast cancer specimens, including Southern, northern and western blots, ELISA, and PCR. However, only two technologies for HER2 status determination are currently validated for use in the routine diagnostic setting: IHC, which identifies HER2 protein expression on the cell surface, and FISH, which determines the degree of *HER2 *gene amplification. Both methods are highly specific and reproducible when performed with a standardised and validated testing protocol.

IHC is far more widely used than FISH. The IHC test for HER2 is semi-quantitative, relating the intensity of the immunostaining to the number of HER2 receptors on the tumour cell's surface. The reagents for the IHC test for the identification of HER2 are relatively inexpensive, the technique is quick, results can be achieved using a conventional light microscope, and the resulting stained slides are easy to preserve and archive. In addition, IHC allows tumour cell morphology to be evaluated by light microscopy. However, variations in methods can affect the results of IHC staining, leading to equivocal results. Such variations can include sample storage conditions, type of fixative used, duration of fixation and type of antibody used. The scoring system for HER2 staining categorises tumours as 3+ (strongly positive staining), 2+ (moderately strong staining), 1+ (weak staining) and 0 (negative for staining). An IHC score of 3+ is classified as positive for HER2 overexpression, but a score of 2+ by IHC is generally considered equivocal; 0 or 1+ are considered HER2 negative. FISH identifies the number of copies of the *HER2 *gene, normally in conjunction with the number of chromosome 17 centromere copies, and is generally seen as being more quantitative than IHC. Furthermore, as DNA is more stable than protein, pre-analytical factors have less impact on test results compared with IHC. However, is more expensive than IHC and takes longer to perform. It also requires special training and access to a fluorescence microscope, which are not available to many laboratories conducting routine diagnostic screening. In addition, the signals produced by the FISH assay decay within a few weeks.

A HER2 testing algorithm has been proposed, and is recommended in most national and international HER2 testing guidelines [18]. Normally, IHC is performed as the initial test to determine HER2 status. Eligibility is clear if the IHC score is either 3+ or 0/1+; women with IHC 3+ tumours are eligible for trastuzumab, whereas those with IHC 0/1+ tumours are not. Women whose tumours produce an equivocal score (IHC 2+) should be retested with FISH; a positive score by FISH is required to confirm eligibility for trastuzumab. However, in cases where FISH is used as the initial test, a positive FISH score indicates eligibility for trastuzumab.

Due to the technical difficulties associated with FISH outlined above, alternative methods of assessing *HER2 *gene amplification status are being investigated. Chromogenic *in situ *hybridisation (CISH) is similar to FISH in that it identifies the degree of *HER2 *gene amplification, and has the same advantages of a DNA-based assay. However, visualisation is achieved using a peroxidase-based chromogenic reaction, similar to IHC. Therefore, unlike FISH, positive signals can be identified using an ordinary light microscope. The signal does not decay, so results can be archived and stored. In recent years, CISH has been evaluated and compared with the established testing standards, IHC and FISH, in a number of studies [19-23]. Most of these studies reported very high rates of concordance (>85%) and thus agreement between these methods. Although it has been shown that when two observers evaluate the same CISH stained slides, inter-observer variability is low [19], inter-laboratory concordance of CISH has not been extensively investigated. To this end, we initiated a ring study involving five international pathology laboratories, to technically validate CISH against the established standard tests for HER2 status and to assess both the intra- and inter-laboratory agreement of CISH.

## Materials and methods

### Tumour specimens

Each laboratory in the ring study provided 40–50 primary invasive breast carcinoma specimens that had been successfully pre-assessed at the original laboratory for HER2 status by both IHC and FISH. Each laboratory included as many cases with low-level amplification assessed by FISH as possible; such cases are relatively rare, but were considered to be of great interest for comparing results of FISH and CISH testing. Each laboratory provided roughly 20 cases scored by FISH as having no amplification, 15 cases scored as achieving high-level amplification, and 6 cases scored as having low-level amplification. Specimens were formalin-fixed (fixation time ranged from 12–48 hours) and embedded in paraffin blocks. Tissue sections for CISH analysis were 4–5 μm, mounted on coated slides.

All specimens were coded for this study; in accordance with the regulations provided by the medical ethical committees from the participating institutes, the data cannot be linked to patient information.

### Assays

The HercepTest (DakoCytomation, Glostrup, Denmark) was used for IHC testing in most of the participating centres; where alternative assays were used, these had been rigorously evaluated using control cases. The results can be considered to be equivalent to those achieved using the HercepTest assay. The PathVysion kit (Vysis, Abbott, Illinois, USA) was used for FISH testing and the SpoT-LIGHT CISH polymer detection kit (Invitrogen, Carlsbad, California, USA) for CISH testing. All assays were conducted as per the manufacturers' instructions or, where TAB250 and CB-11 staining was used, an internally validated and standardised staining protocol.

### Study design

**Figure 1:**
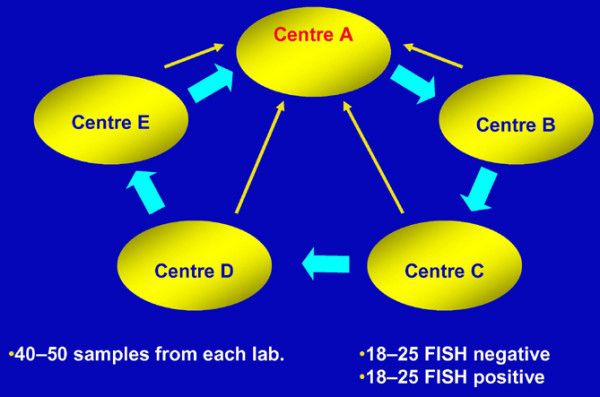
Study design. Each of the 5 centres forward 5 blinded, unstained slides from each sample to the next centre for chromogenic *in situ *hybridisation (CISH) analysis (thick arrows). Results from each centre are reported back to centre A every 2 months (thin arrows).

Five pathology laboratories, each from a different country, participated in the study. Each laboratory conducted IHC, FISH and CISH ('own CISH') on all their samples. Seven to eight slides were prepared for each breast cancer specimen. Five unstained slides from each sample were sent to the next laboratory for CISH analysis ('outside CISH'; Figure 1). The laboratory was blinded to the previous CISH, FISH or IHC results. All scoring results were sent to one laboratory where the correlation between 'outside' CISH and own CISH, FISH and IHC results were calculated.

An additional 15 unstained slides, prepared from one representative breast cancer sample (with areas of HER2-amplified tumour cells and normal tissue) were also sent to the next laboratory in the ring study. These slides were used to conduct a pepsin-time course to determine the optimal digestion time for the sample. The optimal pepsin digestion time was then used in the preparation of the slides tested by CISH.

### Scoring of results

Results for all assays were scored according to the manufacturers' instructions. The results of the IHC tests were scored using the HercepTest scoring system (0–3+). The results of FISH PathVysion kit were scored as follows: a sample was classified as not amplified if it had a HER2/CEP17 ratio of <2.0, low amplified if it had a HER2/CEP17 ratio of 2.0–4.0, and high amplified if it had a HER2/CEP17 ratio of >4.0.

For CISH, the number of HER2 signals per nucleus was counted (at 400× magnification) and categorised as <5 spots, 5 spots, 6 spots or >6 spots (tumours with a large cluster of amplified spots per nucleus were also scored as >6 spots). The scoring system was adapted from the protocol information provided by Invitrogen. At least 30 tumour cells from each specimen were analysed if the sample was deemed homogeneous, but at least 60 cells were analysed if the sample was found to contain between 5 and 10 *HER*2 gene copies in >50% of the area selected for evaluation. The proportion of evaluable tumour cells and the ability to detect normal *HER2 *copy numbers in non-tumour cells were also recorded.

If CISH staining was not successful, this was indicated as 'no signal'.

### Additional quality assessments performed

Heterogeneous quality of the staining result may occur with CISH. To evaluate the extent of heterogeneity in the study results, the percentage of tumour cells with a good CISH result was recorded for each case tested.

A significant advantage that CISH has over FISH is that histology can be examined using a light microscope. Therefore, it was possible to evaluate the morphology of the tumour cells in this study by standard light microscopy. The morphology was categorised as excellent, good, reasonable or poor.

## Results

**Figure 2:**
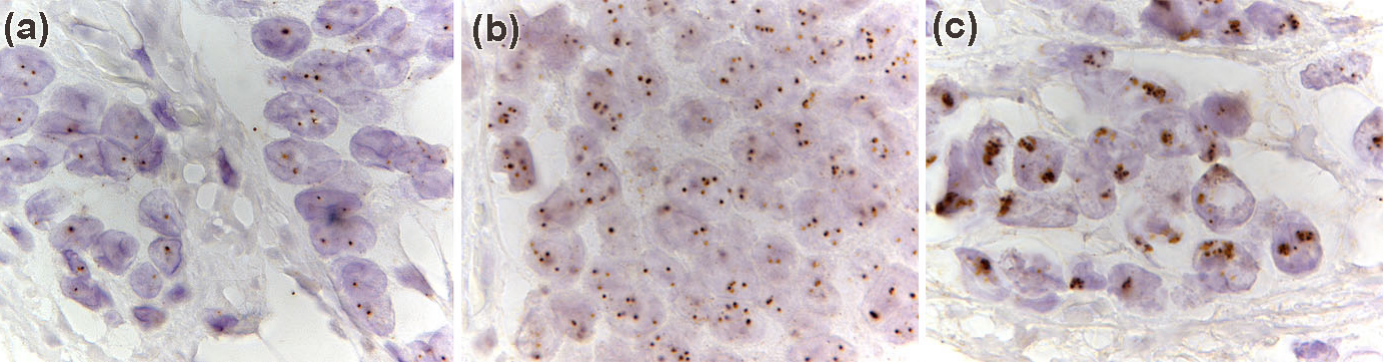
**(a)** chromogenic *in situ *hybridisation (CISH) on a breast carcinoma with normal *HER2 *copy number (1000×). An average of two darkly brown staining copies of the *HER2 *gene can be seen in each tumour cell. **(b)** CISH on a breast carcinoma with low level *HER2 *gene amplification (1000×). An average of 6 copies of the *HER2 *gene can be seen in each tumour cell. **(c)** CISH on a breast carcinoma with high level *HER2 *gene amplification (1000×). Large clusters of darkly brown staining *HER2 *copies can be seen in each tumour cell. It is not possible to count the exact number of *HER2 *gene copies; this is the usual result seen in tumours with high *HER2 *gene amplification.

A total of 211 cases were scored. Typical CISH results are shown (Figure 2). Gene copies can be identified as dots in the nuclei of the cells.

### Inter-laboratory correlation between FISH and CISH

The correlation between the results of FISH testing in the sending laboratory and CISH in the blinded laboratory receiving the unstained slides (outside CISH) is summarised in Table 1. Most cases (70 of 76 cases; 92%) that had a high level of amplification by FISH (HER2/CEP17 ratio of >4.0), were scored as having high amplification by CISH (copy number >6). Three cases (4%) were scored by CISH as having a *HER2 *copy number of 6, indicating a low level of gene amplification. Three more cases (4%) were negative for amplification by CISH; two of these cases contained five *HER2 *gene copies and one contained <5 gene copies as determined by CISH.

**Table 1:** Inter-laboratory correlation between FISH and CISH

	CISH (number of signals)					
		
FISH HER2/CEP17 ratio	<5	5	6	>6	No signal	*n*
<2.0	91	3	0	3	3	100
2.0–4.0	11	4	5	15	0	35
>4.0	1	2	3	70	0	76
						
Total	103	6	8	88	3	211

CISH, chromogenic *in situ *hybridisation; FISH, fluorescence *in situ *hybridisation; HER2, human epidermal growth factor receptor 2.

One hundred of the 211 cases evaluated had previously been shown to have a HER2/CEP17 ratio of <2.0 as determined by FISH, and were HER2 negative. Of these cases, 94 (94%) had ≤ 5 copies of the *HER2 *gene (no amplification) by CISH. Only three of the 100 FISH-negative cases were scored as positive for *HER2 *gene amplification by CISH. In three cases, no signal could be obtained by CISH.

Thirty-five of the 211 cases included in the study were considered low-amplified cases (FISH HER2/CEP17 ratio between 2.0–4.0); of these, 20 cases had an amplified *HER2 *gene copy number by CISH, with five containing 6 *HER2 *gene copies and 15 having >6 copies. In 15 of the 30 cases, CISH did not detect *HER2 *gene amplification and of these, 11 were found to have a *HER2 *copy number <5 and four had a copy number of 5.

### Inter-laboratory concordance of CISH results

Table 2 shows the correlation between CISH performed in the sending laboratory (own CISH) and CISH performed at the receiving laboratory (outside CISH).

**Table 2:** Inter-laboratory correlation between 'own CISH' and 'outside CISH'

	Outside CISH (number of signals)					
		
Own CISH (number of signals)	<5	5	6	>6	No signal	*n*
<5	94	2	1	3	1	101
5	0	1	0	2	1	4
6	3	3	0	3	0	9
>6	4	3	7	78	0	92
No signal	0	0	0	1	1	2
						
Total	101	9	6	87	3	208

CISH, chromogenic *in situ *hybridisation.

Ninety-two cases had a high level of amplification (>6 copies per cell) by own CISH, 85 (92%) demonstrated some degree of *HER2 *gene amplification (≥6 copies per cell) by outside CISH. Seventy-eight of these cases had >6 *HER2 *copies per cell and seven had 6 copies per cell as determined by outside CISH. Seven cases (8%) were classified as negative for amplification (≤5 copies per cell) by outside CISH. Four of these cases had <5 *HER2 *copies per cell and three had 5 copies.

Of the 101 cases that had at least a low-level of *HER2 *gene amplification (≥6 copies per cell) by own CISH, 88 cases also scored positive for amplification by outside CISH (87%), six cases scored 5 copies per cell by outside CISH, and seven cases scored <5 signals per cell.

Most cases (96 of 101 cases; 95%) that scored <5 copies per cell by own CISH, had no amplification (≤5 copies per cell) by outside CISH. Ninety-four of these cases scored <5 *HER2 *copies per cell and two had 5 copies per cell. One case scored 6 *HER2 *copies per cell as determined by 'outside CISH', and three were scored as having >6 copies per cell.

Out of the 105 cases scored as negative for *HER2 *gene amplification (≤5 copies per cell) by own CISH, 97 also scored as negative by outside CISH (92%). One case had 6 *HER2 *copies per cell as determined by outside CISH, and five cases had >6 copies. No signal was detected in a further two cases.

### Intra-laboratory correlation between IHC and CISH

The correlation between IHC and CISH performed in the same laboratory is shown in Table 3. Of the 211 cases included in this study, 86 were scored as HER2 3+ by IHC. Of these, 78 (91%) were scored as having *HER2 *gene amplification by CISH (≥6 copies per cell). Of the 50 cases scored as HER2 0 or 1+, 46 (92%) showed normal *HER2 *copy number by CISH. Of the 75 cases scored as HER2 2+ by IHC, 20 (27%) cases showed *HER2 *gene amplification as assayed by CISH.

**Table 3:** Intra-laboratory correlation between CISH and IHC

	IHC			
		
Own CISH (number of signals)	0/1+	2+	3+	*n*
<5	44	44	6	104
5	2	1	1	4
6	1	5	3	9
>6	2	15	75	92
No signal	1	0	1	2
				
Total	50	75	86	211

CISH, chromogenic *in situ *hybridisation; IHC, immunohistochemistry.

### Intra-laboratory correlation between FISH and own CISH in IHC 2+ cases

According to the current HER2 testing algorithm, FISH is used to confirm IHC 2+ cases. All seven IHC 2+ cases scored as having high levels of amplification by FISH were also scored as highly amplified (>6 copies) by CISH (100%) (Table 4). Of the 47 IHC 2+ cases scored as not amplified by FISH, 44 (94%) were negative by CISH (≤ 5). Of 18 IHC 2+ cases with low amplification by FISH, 12 were negative by CISH (11 had <5 *HER2 *copies per cell, one had 5 copies per cell), two had 6 copies per cell, and four were scored as having >6 copies per cell.

**Table 4:** Correlation between FISH and own CISH in IHC 2+ samples

	Own CISH (number of signals)				
		
FISH	<5	5	6	>6	*n*
<2.0	44	0	3	0	47
2.0–4.0	11	1	2	4	18
>4.0	0	0	0	7	7
					
Total	56	1	5	11	73

CISH, chromogenic *in situ *hybridisation; FISH, fluorescence *in situ *hybridisation; IHC, immunohistochemistry.

### Other quality parameters of the CISH test

Good CISH staining was seen for 75–100% of cells in 65% of the cases examined, 50–75% of cells in 17% of cases, 25–50% of cells in 11% of the cases and 0–25% of cells in 7% of cases.

Tumour cell morphology was found to be excellent in 15.5% of cases, good in 67% of cases, and reasonable in 15% of cases (Table 5). Poor tumour cell morphology was observed in only 2.5% of cases.

**Table 5:** Tumour cell morphology

	Morphology	
Excellent	33/214	15.5%
Good	144/214	67.0%
Reasonable	32/214	15.0%
Poor	5/214	2.5%

## Discussion

Accurate assessment of HER2 status is essential for identifying patients who are candidates for therapy with the anti-HER2 monoclonal antibody trastuzumab. A rapid and accurate test for HER2 status is needed because an increased number of treatment decisions are based on the HER2 status of a patient's disease. In particular, increasing evidence suggests that response to certain forms of hormone treatment and chemotherapy are dependent on a patient's HER2 status [9-14]. The results of our multicentre ring study show that CISH is a reliable test for the assessment of *HER2 *gene copy number.

The study demonstrates excellent correlation between FISH and CISH results, with 92% of cases scored as having high-level amplification by FISH also scoring as having high-level amplification by CISH. In addition, 94% of cases which were shown to have a normal *HER2 *gene copy number by FISH appeared to lack *HER2 *gene amplification by CISH. Inter-laboratory concordance with CISH was similar to that seen between FISH and outside CISH; 94% for ≤5 *HER2 *copies per cell and 92% for ≥6 *HER2 *copies per cell.

Intra-laboratory correlation of IHC with CISH was also good for cases scored as 3+ and 0 or 1+ by IHC (91% and 92% of cases respectively). Twenty (27%) of the 75 cases scored as HER2 2+ by IHC showed *HER2 *gene amplification as assayed by CISH. This is in agreement with the findings of other studies comparing HER2 overexpression with the degree of *HER2 *gene amplification. In these studies, the percentage of tumours with a HER2 IHC 2+ score and gene amplification was approximately 25% [12,25,26].

Accurate scoring of cases with low-level *HER2 *gene amplification is technically challenging. Therefore, a high number of cases with a HER2/CEP17 ratio of 2.0–4.0 were specifically included for evaluation in this study. According to the FISH scoring guidelines, these cases are positive for *HER2 *gene amplification. However, 43% of cases scored as low level of amplification by FISH, but scored as non-amplified by CISH. Further detailed analyses should be conducted in this area to clarify this result. However, it should be noted that breast carcinomas with a low degree of *HER2 *gene amplification are rare (estimated to be 1–3% of all carcinomas), and inter-observer variability is greatest in this category of samples. Indeed, three out of seven cases with low-level amplification assessed by FISH included in this study gave different results when re-assessed by FISH in another laboratory (data not shown). A recent inter-observer study for FISH showed that, although agreement was excellent for tumours with normal *HER*2 gene copy numbers or *HER*2 gene amplification, there was marked inter-observer variability in these 'borderline cases' [27]. Recently, a consensus panel has proposed adapted scoring guidelines for HER2 testing. An important recommendation from this panel was to consider reporting breast cancer cases with a HER2/centromer chromosome 17 ratio between 1.8 and 2.2 as borderline [28]. Using this adapted scoring guideline, a tumour is assessed as *HER2 *amplified when the ratio is more than 2.2; or when the absolute number of *HER2 *gene copies is more than six.

The key to understanding FISH/CISH discrepancy in cases with low-level amplification lies in the nature of the two tests. A significant difference between FISH and CISH is that in the most commonly used FISH assay, 2-colour FISH, the copy number of the *HER2 *gene and of the centromere of chromosome 17 (CEP17) are assessed simultaneously. The signal from the centromere probe functions as an internal control; the final FISH score is based on a ratio of the signals from the two probes. Conversely, in the CISH assay, the copy number of *HER2 *is assessed directly. When classifying tumours with one to four copies, or with >10 copies of *HER2 *by CISH, the *HER2 *status of the sample is clear. However, in cases with *HER2 *copy numbers in the range of 4–10, the HER2/CEP17 ratio may be of importance. Some discrepancies between FISH and CISH may also be explained for polysomic cases where the FISH assay might indicate a negative testing result, as the HER2/CEP17 ratio is calculated, while CISH indicates a *HER2*-positive testing result as the numbers of HER2 signals are assessed. In general, we have also noted that the inter-observer variability in scoring cases with mid-range *HER2 *copy numbers is relatively large, and also that inter-observer variability in scoring HER2/CEP17 ratio with FISH is considerable (unpublished results). According to the Invitrogen scoring system for CISH, supplied by the manufacturer at the start of this study, a score of ≤5 *HER2 *copies per cell indicates no amplification, whereas a score of ≥6 *HER2 *copies indicates amplification. This meant that classification of cases with an average CISH score of 5–6 was unclear. However, since this study, Invitrogen has updated its scoring system to specify that a score of >5 signals per cell should now be considered indicative of *HER2 *gene amplification. As we wished to be able to categorize the subgroups in this borderline category more precisely, scoring 5 copies and 6 copies as separate categories was part of the study design. We believe that obtaining a reliable CISH result for these borderline cases is no more problematic than obtaining a reproduced FISH result for such cases. A practical approach to this problem is to count at least 60 nuclei when the number of *HER2 *copies ranges from 4–10, and to consider adding CISH using a CEP17 probe on a consecutive slide to confirm the result. Alternatively, and where possible, dual-colour FISH with probes for HER2 and CEP17 can be considered. It is to be hoped that it will be possible to analyze the studies showing benefit of trastuzumab therapy in the adjuvant setting for the benefit of the subgroup of *HER*2 low-level/borderline amplified tumours. This may help to better define the analytical approach to this category of tumours.

When own CISH was compared with FISH in determining the HER2 status of IHC 2+ cases, there was 100% concordance for HER2-positive samples and 94% for HER2-negative samples. The good intra-laboratory correlation of FISH with CISH suggests that CISH could potentially be used in place of FISH to determine the HER2 status of equivocal cases by IHC. As both tests produce similar results in the retesting of equivocal IHC cases, CISH could potentially fulfil the same role as FISH in the HER2 testing algorithm.

CISH displayed high inter-laboratory concordance, showing that results are reproduced between laboratories. Correlation between own CISH and outside CISH was similar to that observed between FISH and outside CISH with 95% concordance for cases with <5 *HER2 *copies per cell and 92% for cases with >6 *HER2 *copies per cell. The most notable discrepancies with CISH in a few cases are probably the result of technical problems with the staining procedure or with the interpretation of the results. This highlights the need for training before any technology is used for the first time. However, the excellent inter-laboratory correlation of CISH results highlights its potential as a method for testing HER2-status, even in those laboratories unfamiliar with using CISH.

As can be seen from our results, this study includes cases with a HER2 IHC3+ score but without *HER2 *gene amplification (as detected by CISH); and cases with a HER2 IHC0/1+ score and with *HER*2 gene amplification (as detected by CISH). The number of discrepancies was very similar if FISH was used instead of CISH (data not shown). In view of the clinical consequences of accurate HER2 status assessment for many patients, it should be considered to test CISH (or FISH) alongside IHC either as a diagnostic routine or as part of a quality assurance program in each lab.

The pre-treatment of tissue sections, especially the pepsin digestion time, was a critical step in achieving a good CISH result. Like FISH, the optimal pepsin digestion time differs between tumours. For practical reasons, the pepsin digestion time used in the preparation of received slides was calculated in each laboratory on the basis of a pepsin time course performed on one representative tumour provided by the sending laboratory. However, it is possible that this was not the optimal value for all tumour samples, and as a result it was likely that in some cases no HER2 signal would be detected. For such cases, it was determined that the CISH test should be repeated after adjustment of the pepsin digestion time. Using this strategy, a good result was obtained in 99% of the cases (208 out of 211). In all cases, the percentage of tumour cells for which *HER*2 copy number could be assessed by CISH was recorded; this percentage was >50% for most cases (data not shown). There were no cases in this series where noticeable heterogeneity in the number of *HER*2 signals per tumour cell was observed.

An advantage of CISH over FISH is that invasive tumour cells can be easily identified using a light microscope, provided that the morphology of the tissue is good following CISH staining. More than 80% of the tumours examined to date in this study had either good or excellent morphology, illustrating this beneficial aspect of CISH testing.

## Conclusion

**Figure 3:**
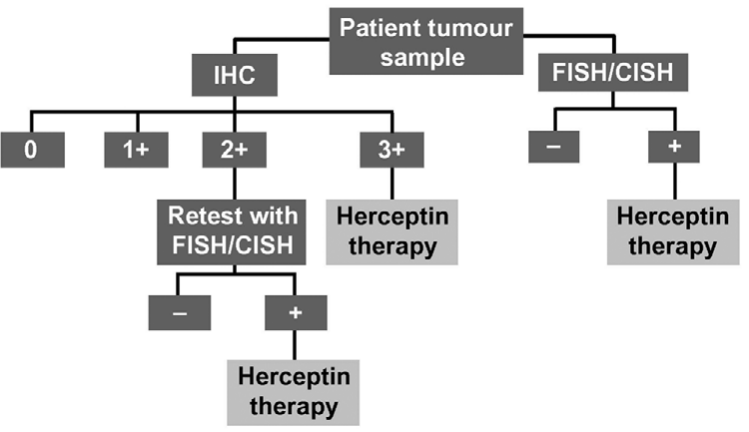
The recommended HER2 testing algorithm. Reprinted with permission from W Hanna.

This validation ring study demonstrates that CISH is a very powerful test for the determination of *HER2 *gene copy numbers in breast cancer specimens. Moreover, CISH also has significant advantages over FISH, which is considered to be the reference standard for determination of *HER2 *gene amplification. These results are similar to the findings of other studies that have evaluated CISH [19-24]. The concordance of CISH with FISH in this study is especially encouraging as CISH tests were performed in a different laboratory to the one in which the tumour sections were prepared, which adds a greater level of significance to these trial results. Therefore, in view of these results, we suggest that CISH can be used as an alternative method to FISH in the current HER2 testing algorithm (Figure 3).

## Abbreviations

CISH = chromogenic *in situ *hybridisation; FISH = fluorescence *in situ *hybridisation; IHC = immunohistochemistry; HER2 = human epidermal growth factor receptor 2.

## Competing interests

MvdV, MB, WH, F P-L and JR are members of the F Hoffmann-La Roche AG Herceptin Pathology Advisory Board and are payed honoraria for attendance at these meetings. MH declares that he has received honoraria from F Hoffmann-La Roche AG for teaching about the assessment of HER2 on several occasions in recent years. PK declares that she has no competing interests.

## Authors' contributions

Each of the authors was involved in the design of the study; in overseeing the staining of the slides; and in interpreting the staining results. MvdV and PK were involved in analyzing the data. All the authors were involved in drafting the manuscript.
